# Serotypes of group B streptococci in western Sweden and comparison with serotypes in two previous studies starting from 1988

**DOI:** 10.1186/s12879-015-1266-4

**Published:** 2015-11-09

**Authors:** Margrét Johansson Gudjónsdóttir, Elisabet Hentz, Stefan Berg, Erik Backhaus, Anders Elfvin, Samir Kawash, Birger Trollfors

**Affiliations:** Division of Neonatology, Department of Paediatrics, Institute of Clinical Sciences, Sahlgrenska Academy, Gothenburg University, Gothenburg, Sweden; Department of Pediatrics, Queen Silvia Childrens’ Hospital, Sahlgrenska University Hospital, Gothenburg, Sweden; Department of Infectious Diseases, Skaraborg Hospital, Skövde, Sweden; Department of Bacteriology, Sahlgrenska University Hospital, Gothenburg, Sweden; Department of Pediatrics, Neonatology, Sahlgrenska University Hospital, 41685 Gothenburg, Sweden

**Keywords:** Group B Streptococci, Serotypes, Invasive infections, Neonates, Streptococcus agalactiae, Epidemiology

## Abstract

**Background:**

Group B Streptococci (GBS) are the most common neonatal pathogens and infect immunocompromised and elderly individuals. The species has 10 different serotypes. Serotypes have been studied in the south-west area of Sweden in 1988–1997 and 1998–2001. The aim of this study was to study serotypes in the same area from 2004 to 2009.

**Methods:**

Invasive GBS isolates were collected prospectively from 2004 to 2009 in two counties in western Sweden with a population of 1.8 million, and were serotyped by latex agglutination. Clinical data were obtained from hospital records. During the study period 410 invasive GBS isolates from 398 patients were collected (multiple episodes ≥1 month apart). Clinical data were not available for two patients who are excluded. Four isolates were from stillborn neonates, 88 were from live born neonates and infants, and 318 from adults.

**Results:**

Serotype III was the most common serotype (48 %) in neonates and infants followed by serotypes Ia (18 %) and V (16 %). In adults serotype V (39 %) dominated followed by serotypes III (20 %) and Ib (14 %). There was a significant increase of serotype V in comparison with the first study (1988–1997) but there were no significant changes in the serotype distribution between the present study and the second study (1998–2001). There were a few cases of serotype VI-IX, both in children and adults, not seen in the previous studies. Serotype V was more common among patients with arthritis than with any other manifestation.

**Conclusions:**

Changes in GBS serotypes occur over time in the same region, which must be considered when GBS vaccines are formulated.

## Background

Group B Streptococci (GBS) remain a leading cause of invasive neonatal infections and an important cause of infections in pregnant women, immune compromised adults and the elderly [1–3]. GBS possess ten distinct capsular polysaccharide (CPS) types: Ia, Ib and II-IX [4]. The principal disease-associated serotypes are currently Ia, Ib, II, III and V [2, 5–7] according to studies from North America and Norway.

Most centers in Sweden have been using a risk-based prophylaxis strategy which identifies candidates for intra partum antimicrobial prophylaxis (IAP) according to the presence of any of the following risk factors: a previous GBS-infected baby, positive for GBS in urine, delivering at <37 weeks’ gestation, having an intra-partum temperature ≥38.0°, or rupture of membranes for ≥18 h. There is no plan to begin with universal prenatal GBS screening followed by IAP of all GBS carriers as is recommended by the US guidelines from 2002 [8, 9]. Both strategies have dramatically reduced the incidence and mortality among early onset (EO, <7 days after birth) GBS infections, but they have not reduced the incidence of late onset (LO, 7–27 days after birth) or very late onset (VLO, 28 days–4 months after birth) disease [1, 10–13].

There are no efficacy studies of GBS vaccines, but most likely protection would be achieved by anticapsular serum antibodies as it is for other bacteria with polysaccharide capsules. The first vaccines against GBS consisted of pure polysaccharides and had disappointingly low immunogenicity. Polysaccharide-protein conjugates are more immunogenic and have been given in phase 1 to 2 clinical trials (including pregnant women with 1 to 5 serotypes. Proteins used have been tetanus toxoid or genetically inactivated diphtheria toxoid [14]. To make the best possible GBS vaccines they must be directed against the polysaccharides of the most common GBS serotypes.

It is important to follow the distribution of serotypes since it may differ over time and between populations [3].

The main aim of this study was to survey the current serotype distribution of invasive GBS infections in a Swedish population and to compare it with previous studies in the same area to detect any changes over time.

## Methods

Invasive GBS isolates were collected prospectively between 2004 and 2009 from the six bacteriological laboratories, which served all 13 hospitals, in the counties of Västra Götaland and Halland in western Sweden. The mean population of the surveillance area was 1,828,140 which corresponds 20 % of the total population in Sweden (9,128,308 inhabitants). The total number of live births was 127,882 during the study period. (Central Bureau of Statistics, Stockholm, Sweden (http://www.scb.se). The two regions studied include the city of Gothenburg with suburbs, (urban area population about 850,000, cities with populations up to 100,000 inhabitants, small villages and rural areas. There are no major socioeconomic differences within the area studied nor between the area studied and the rest of Sweden.

An invasive GBS case was defined as isolation of the organism from a normally sterile site (blood, cerebrospinal fluid and synovial fluid) in a surveillance area resident. No isolates came from pleural, pericardial or peritoneal fluid*.*

The total number of invasive GBS isolates during the time period was 515. Thus of all isolates 410/515 (80 %) were serotyped. The strains which were not serotyped were evenly distributed during the time period and the participating laboratories. Age, date of culture, possible risk factors, and complications, were obtained from medical records and an evaluation was made to see if death was attributed to the GBS infection. One neonate had protected identity, so its records could not be studied. The records of one adult patient could not be found. These two patients are excluded from all parts of the study.

Isolates were identified as GBS by colony morphology, microscopy following Gram’s stain of smears, and co-agglutination with group-specific reagents (Streptex; Murex Biotech, Dartford, UK). The isolates were stored in a broth at −70 °C until serotyping was performed using the latex agglutination test with type-specific antisera for serotypes Ia, Ib, II, III, IV, V, VI, VII, VIII and IX (Statens Serum Institut, Copenhagen, Denmark), as previously described [15].

Only one isolate from each infectious episode was included in the study and it was considered the same episode if it was less than 30 days between the isolates. GBS was isolated from 410 infectious episodes in 398 patients, 11 patients had more than one episode. Each infectious episode is reported as a separate patient.

### Statistics

Fisher’s exact test (two-tailed) was used for comparisons of proportions (http://graphpad.com/quickcalcs/contingency1.cfm). A *p* value <0.05 was considered significant.

### Ethical approval

The study was approved by the Ethics Committees of Gothenburg University and Lund University (registration numbers Ö524-03, LU674-02). The committees did not require individual consent, because the clinical data were obtained in retrospect, when many patients and/or relatives could not be found.

## Results

### Neonates and infants

A total of 91 invasive GBS isolates from 91 neonates and infants aged 0–209 days were serotyped, 47 boys and 44 girls. Four were intra uterine fetal deaths (IUFD). Sepsis without a known focus was the most common clinical manifestation among live born neonates and infants with 63 cases (72 %). Meningitis was seen in 12 patients (14 %). Other manifestations were pneumonia (eight patients), skin infection (two patients), urosepsis (one patient) and septic arthritis (one patient). Altogether 33 (38 %) live-born patients were preterm, i.e. gestational age <37 weeks, 20 had EO disease, two had LO disease and 11 had VLO disease.

**Fig. 1:**
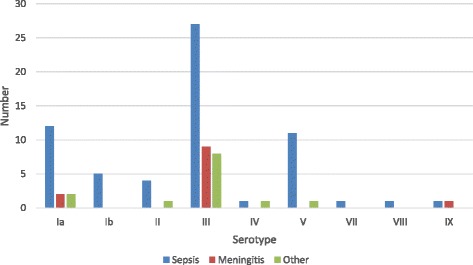
Serotype distribution among live-born neonates and infants (*n* = 87) with different manifestations. *Other: Pneumonia (8), Erysipelas (2), Urosepsis (1), Septic Arthritis (1)

Serotype III was the most prevalent serotype (48 %) in neonates and infants followed by serotype Ia (18 %) and serotype V (16 %). The serotype distribution compared to clinical manifestations is shown in Fig.1. Serotype III was more common (*p* = 0.03) in patients with sepsis with no known focus (IUFD not included) than the other serotypes. There were no other significant differences related to clinical manifestations and serotypes.

A total of seven children died because of invasive GBS infection; all had sepsis, three were born full term and four were born prematurely. A total of five were EO, one VLO and one was 122 days old. They had a variety of serotypes with no one dominating.

### Adults

There were 317 adults (median age 73 years (23–103 years) 170 males and 147 females). Underlying medical conditions were documented in 259 (81 %), the most common being cardiovascular disease (*n* = 114), diabetes (*n* = 80) and malignant disease (*n* = 62).

Serotype V was the most prevalent serotype (39 %) followed by serotypes III and Ib (20 and 14 %, respectively) (Table 1). There were a few significant relations between serotype and clinical manifestation. Serotypes V and III were more common among patients with septic arthritis than with any other manifestation (*p* = 0.008 and *p* = 0.03 respectively). Otherwise there was no difference in the serotype distribution related to clinical manifestations (Table 2).

**Table 1:** Serotype distribution among invasive group B streptococci

	Neonates and infants						Adults		Total	
	IUFD^a^		Early onset		Late and very late onset					
Serotype	No.	(%)	No.	(%)	No.	(%)	No.	(%)	No.	%
Ia	1	(25)	12	(20)	4	(13)	34	(11)	51	(12)
Ib			5	(9)	0	(0)	45	(14)	50	(12)
II			3	(5)	2	(7)	29	(9)	34	(8)
III	3	(75)	24	(41)	20	(67)	62	(20)	109	(27)
IV			1	(2)	1	(3)	14	(4)	16	(4)
V			11	(19)	1	(3)	124	(39)	136	(33)
VII			1	(2)	0	(0)	0	(0)	1	(<1)
VIII			0	(0)	1	(3)	3	(1)	4	(1)
IX			1	(2)	1	(3)	7	(2)	9	(2)
Total	4		58		30		318		410	

^a^Intra Uterine Fetal Death

**Table 2:** Serotype distribution of invasive group B streptococci related to clinical manifestation in adults

	Serotype								
Manifestation	Ia	Ib	II	III	IV	V	VIII	IX	Total (%)
Sepsis, unknown focus	11	13	9	20	5	32	0	2	92 (29)
Erysipelas	12	13	7	9	1	30	2	1	75 (24)
Septic arthritis*	3	6	3	8	2	29	0	1	52 (16)
Urosepsis	3	6	4	12	2	12	0	2	41 (13)
Pneumonia	1	2	3	4	2	9	1	0	22 (7)
Endocarditis	2	3	1	5	1	7	0	1	20 (6)
Meningitis	1	0	0	3	0	0	0	0	4 (1)
Other^a^	1	2	2	1	1	4	0	0	11 (3)
Missing clinical data	0	0	0	0	0	1	0	0	1 (0,3)
Total (%)	34 (11)	45 (14)	29 (9)	62 (20)	14 (4)	124 (39)	3 (1)	7 (2)	318

*p = 0.008 and *p* = 0.03 when the proportion of arthritis was caused by serotype V and III respectively was compared with the proportion of arthritis for all other serotypes

^a^Chorioamnionitis, Mediastinitis, Periotinitis, Cholecystitis, Vulvitis

During the study period 38 patients (12 %) died during or within 30 days after the infection, 20 (53 %) had sepsis with unknown focus. Other manifestations were erysipelas (five), pneumonia (four), endocarditis (three), urosepsis (three), meningitis (one), septic arthritis (one), and mediastinitis (one). Only five of the patients who died did not have a known underlying medical condition, three were older than 72 years, one woman at age 53 died in multi organ failure 2 days after diagnosis of sepsis, one woman died at the age of 24 because of pneumonia, she had given birth 17 days prior to death. The serotypes isolated from the patients who died were V (12), III (ten), Ia (five), II (five), Ib (three), IV (two) and IX (one). No difference in serotype distribution could be seen among the fatal infections and in patients who survived.

### Recurrent or reinfections

A total of 11 patients, all adults with a preexisting underlying condition, had more than one episode at intervals from 2 to 23 months. The second infection was caused by a different serotype in six patients and by the same serotype in 5 patients (Table 3). The clinical manifestations varied but were the same in both (or all three) second infections in 8 patients.

**Table 3:** Patients with more than one invasive isolates of group B streptococci

Patient no.	Age (months)	Serotype	Manifestation	Time between infections (months)	Underlying condition
1	81	Ib	Septic arthritis	9	Prosthetic hip, malignancy
	81	Ib	Septic arthritis		
2	79	V	Septic arthritis	3	Prosthetic hip
	79	V	Septic arthritis		
3	77	III	Urosepsis	2	Chronic urinary catheter malignancy, dementia
	77	Ib	Urosepsis		
4	73	V	Sepsis, unknown focus	4	Heart valve operation
	73	V	Mediastinitis		
5	67	Ia	Spondylitis	2	Spinal stenosis
	68	Ia	Spondylitis		
6	62	II	Erysipelas	10	Progressive multiple sclerosis, urostomy
	63	II	Erysipelas		
7	60	V	Septic arthritis	8	Ischaemic Heart Disease
	60	V	Endocarditis		
8	61	V	Erysipelas	8	Malignancy. Died after the skeptical infection
	62	IV	Sepsis, unknown focus		
9	46	V	Urosepsis	23	Chronic urinary catheter
	48	II	Urosepsis		
10	33	V	Septic arthritis	22	Chronic renal failure, urostomy
	34	II	Septic arthritis		
11	82	V	Erysipelas	10	Malignancy
	83	V	Erysipelas	25	
	85	III	Erysipelas		

### Differences between children and adults

The major difference between children (neonates and infants) and adults was that serotype III was much more common among children (*p* < 0.0001) and serotype V among adults (*p* < 0.0001). Serotype Ib was also more common among adults than children (*p* = 0.029).

### Comparison of the present study with two earlier studies from the same area

The main aim of this study was to survey the serotype distribution of invasive GBS infections and to compare it with previous studies in the same area to detect any changes over time.

Table 4 compares the serotype distribution with results from previous studies in the same region [16, 17]. There was a significant increase of serotype V between the first study (1988–1997) [16] and the second study (1998–2001) (*p* = 0.007 when serotype V was compared with all other serotypes) [17]. Serotype V has remained on approximately the same high level among adults in the present study. Serotype III has been the most prevalent serotype isolated from neonates and infants in all three studies from south-west Sweden since 1988 [16, 17, present study]. Serotypes Ia, Ib, II, III and IV have shown some fluctuations between the studies, but all differences were non-significant and showed no obvious trends over time. There were a few cases of serotype VII-IX, both in children and adults, which have not previously been seen in this area. Serotype VI has remained absent.

**Table 4:** Serotype distribution of invasive group B streptococci isolates in the present study compared with two previous studies in the same region

	Neonates and infants						Adults					
	Berg et al. [15]		Persson et al. [16]		Present study		Berg et al. [15]		Persson et al. [16]		Present study	
Serotype	No.	(%)	No.	(%)	No.	(%)	No.	(%)	No.	(%)	No.	(%)
Ia	14	(18)	5	(10)	17	(18)	4	(6)	10	(9)	34	(11)
Ib	2	(3)	2	(4)	5	(6)	15	(23)	10	(9)	45	(14)
II	4	(5)	1	(2)	5	(6)	10	(15)	7	(6)	29	(9)
III	48	(62)	30	(60)	44	(48)	19	(29)	28	(25)	62	(20)
IV	2	(3)	1	(2)	2	(2)	1	(2)	8	(7)	14	(4)
V	7	(9)	11	(22)	15	(16)	14	(21)	47	(42)	124	(39)
VII	0	(0)	0	(0)	0	(0)	0	(0)	0	(0)	1	(1)
VIII	0	(0)	0	(0)	3	(1)	0	(0)	0	(0)	1	(1)
IX	0	(0)	0	(0)	7	(2)	0	(0)	0	(0)	2	(2)
Non-typeable	1	(1)	0	(0)	0	(0)	3	(5)	1	(1)	0	(0)

The present study covers a longer time span than the two previous, which explains the increased number of cases

## Discussion

The differences in serotype distribution between neonates/infants and adults must be considered in future GBS vaccines. Pregnant women are the obvious target for GBS vaccination in order to protect neonates from early onset disease through passively transferred antibodies, so (at least currently) the most important in a vaccine must be type III followed by serotype Ia while serotype V seems to be of little importance. A GBS vaccine for elderly or patients with severe underlying diseases, in which serotype V infections are the most common, is probably unrealistic. There has been little change in serotype distribution among neonates and infants over the last 30 years, even after the rise of serotype V among adults [5]. Serotype V was first reported in 1985 and soon became the most common serotype among adults in many parts of the world [17–19].

The present study showed that five serotypes, Ia, Ib, II, III and V, accounted for 94 % (neonates and infants) and 93 % (adults) of cases with invasive GBS infections. This is consistent with studies from other countries [2, 5, 20, 21]. Serotype III is still the most common serotype among neonates and infants, especially for LO disease [5, 11, 13, 19, 20, 22]. Other studies have shown a dominance of serotype Ia for EO disease [19, 23, 24]. A possible reason for this differences between different parts of the world could be that GBS has similarities with pneumococci. It is well known that serotypes of pneumococci change regionally and over time [25, 26]. This was seen already in the prevaccination era. One example is the increase in pneumococcal serogroup 1 in Northern Europe [27–29]. A reason for this could be that pneumococci have different clones with different virulence within the same serotype. This has been suggested to be the case in increase of pneumococcal serogroup 1. Possibly this may be true also for GBS.

It has previously been suggested that a trivalent vaccine has the potential to further reduce the burden of GBS disease and especially LO cases [30]. Regarding our findings a trivalent vaccine (CRM197-conjugated capsular polysaccharides of GBS serotypes Ia, Ib and III), could have had an impact on 80 % (24/30) of the LO cases and 75 % (69/92) of all infants and neonates in our population during the study period. Several studies have shown serotype replacement among pneumococci when pneumococcal conjugate vaccines have been used in large scale for some years [31–33]. Only future can tell if this will occur also among GBS if such vaccines are used extensively.

Such a vaccine would have little impact on GBS diseases in adults, e.g. those with severe underlying diseases. Among adults three serotypes, Ia, III and V account for 70 % of cases with invasive GBS infections. Other studies have reported similar findings (64–71.8 %) but with variation in which of the three is the most prevalent [2, 18, 20, 22, 34, 35].

Some studies have reported an increase in serotype IV among invasive GBS isolates [36, 37]. We did not see any rise of serotype IV in the present study in comparison with the two previous studies done in the same region [16, 17]. Other studies show like ours that these serotypes are nonexistent or very uncommon [22, 23].

There were few relations between serotype and clinical manifestations but the study showed that serotypes V and III were found more often isolated from synovial fluid in adults but others have found it to be serotype II [38].

## Conclusions

In conclusion, this study has shown that there have not been any significant changes of the serotype distribution of invasive GBS infections in this Swedish population since the emergence of serotype V in the late 1990s but a few cases of serotypes VII-IX, which are not being targeted with a vaccine, have emerged in the area. It is therefore important to have an ongoing surveillance of GBS even though a trivalent vaccine would probably lead to much less disease among neonates and infants.

## Abbreviations

GBS: Group B Streptococci
CPS: Capsular polysaccharide
IAP: Intra partum antimicrobial prophylaxis
EO: Early onset
LO: Late onset
VLO: Very late onset
IUFD: Intrauterine fetal death
